# Effect of Sex Differences in Silicotic Mice

**DOI:** 10.3390/ijms232214203

**Published:** 2022-11-17

**Authors:** Fuyu Jin, Yaqian Li, Xiaojing Wang, Xinyu Yang, Tian Li, Hong Xu, Zhongqiu Wei, Heliang Liu

**Affiliations:** Hebei Key Laboratory for Organ Fibrosis Research, School of Public Health, North China University of Science and Technology, Tangshan 063210, China

**Keywords:** silicosis, mouse sex, collagen, inflammatory response, senescence, fibrosis

## Abstract

Mechanisms of silicosis, caused by the inhalation of silica are still unclear, and the effect of sex on silicosis has rarely been reported. The purpose of this study was to investigate whether sex affects the silicotic lesions and the progressive fibrotic responses in silicosis. Our study showed that sex had no significant effect on the area of silicon nodules and the collagen deposition after a one-time bronchial perfusion of silica. Immunohistochemical staining showed that CD68 and the transforming growth factor-β1 (TGF-β1) were positive in male and female silicotic mice. In addition, the western blot results showed that the fibrosis-related factors type I collagen (COL I), α-smooth muscle actin (α-SMA), vimentin, TGF-β1, p-SMAD2/3, inflammatory-related factors interleukin 6 (IL 6), interleukin 1β (IL 1β), and senescence-related factors p16 and p21 were up-regulated in silicotic mice and there was no difference between female or male mice exposed to silica. The expression of TGF-β1, p-SMAD2/3, p16, and p21 were downregulated in the early stage of female silicotic mice, compared to the males. Thus, despite differences in the expression of certain factors, there was no overall difference in the progressive fibrosis between female and male mice in silicosis. These results thus provide a new perspective for studying the pathological development of silicosis.

## 1. Introduction

Pneumoconiosis, caused by the inhalation of silica (SiO_2_), is the most common occupational disease in China, accounting for about 80% of the total number of occupational diseases reported every year [1]. However, due to the low coverage of occupational health examinations and the strict diagnostic criteria for pneumoconiosis, the number of cases is underestimated [2]. The mechanisms of silicosis are not very clear yet, and thus effective treatments are still lacking, relying on lung transplantation [3].

The pathogenesis of silicosis has been addressed in vivo and in vitro for many years [4,5,6,7] Factors, such as inflammatory response, transforming growth factor-β (TGF-β) signaling, and senescence-related factors [4,5,7,8,9], play important roles in the progression of experimental silicosis. Most studies performed were on male mice or rats. 

In several studies related to the occupational environments dominated by men, often there is no distinction by sex or women are excluded from the analysis. It is this chronic neglect that has led to the default choice of male subjects in most studies. In recent descriptions, nearly 100% of coal workers with advanced pneumoconiosis are men [10,11] Similarly, in large epidemiologic surveillance programs for silicosis across the United States, more than 95% of cases were found in men [12]. From a few available epidemiological data on the sex distribution of silicosis patients, it is observed that even if the number of female patients is small, it is undeniable that there is a female group among the silicosis patients. Thus, it is not possible to theorize that either men or women are more susceptible to the effects of silica. Therefore, to provide a theoretical basis for the pathological study and the clinical treatment of silicosis, it is necessary to investigate the effect of the sex differences on silicotic animal models.

Sex has also been reported as a critical factor in determining the susceptibility toward various agents that cause acute or chronic respiratory inflammation in human pulmonary diseases [13,14]. A study about the sex differences in the incidence and outcomes of idiopathic pulmonary fibrosis (IPF) patients showed that in elderly patients, the incidence of IPF was higher among men than women [15]. Similarly, in murine models of chemically induced pulmonary fibrosis, female mice were protected from the chronic complications with a single exposure to either hydrochloric acid or nitrogen mustard [16]. In a similar study, Dorothy et al. found that male mice were more susceptible to a single acute or repeated sub-chronic exposure to nickel nanoparticles, in the absence or presence of lipopolysaccharide [17]. All of these studies thus suggested that sex difference seems to play a significant role in the progression of pulmonary diseases. Based on the existing research, the present study determined the effect of sex on experimental silicosis mice and provided a new perspective for studying the pathological development of silicosis in the future.

## 2. Results

### 2.1. Effects of Sex on the Silicotic Nodules and Pulmonary Functions

**Figure 1:**
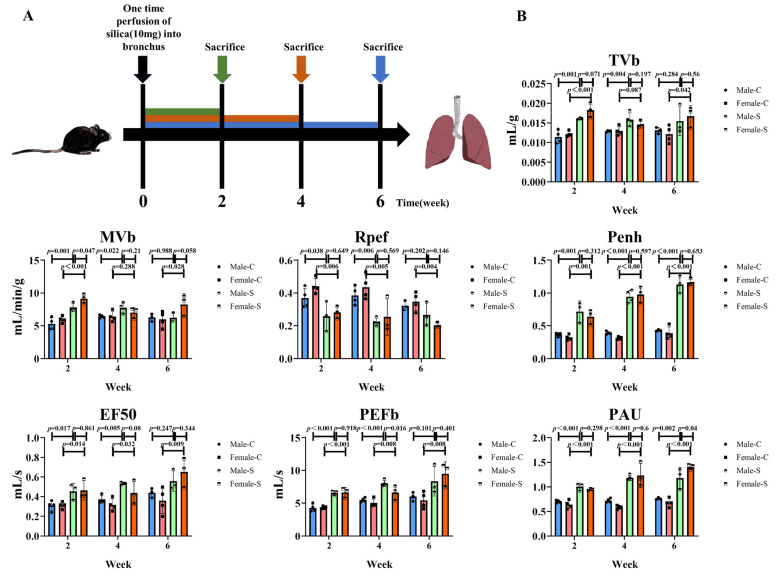
Indexes of the pulmonary function of male and female mice exposed to silica in two, four, and six weeks, and the control groups. (**A**) Schematic diagram of a silicotic model establishment and execution. (**B**) Detection indexes of a lung function, *n* = 5 per group.

**Figure 2:**
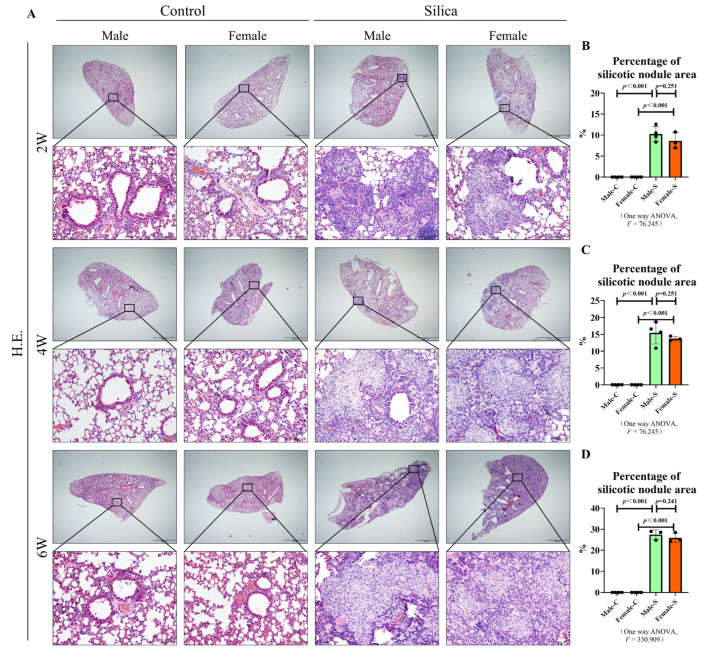
H&E staining of male and female mice exposed to silica in two, four, and six weeks, and the control groups. (**A**) H&E staining of the lung tissues in mice exposed to silica (scale bar = 2000 µm). (**B**–**D**) Statistical analysis of the H&E staining, *n* = 5 per group ((**B**)—2W, (**C**)—4W, (**D**)—6W).

To determine the effects of sex on the silicotic nodules, the silicosis models were established by injecting silica into the trachea of male and female mice. The mice were further sacrificed at different time points (Figure 1A). The death rate of the mice was 3.3%, and the average weight of the mice in the control group increased slightly with the prolongation of time (male: 20-22-23-25 g; female: 16-17-20-22 g), while the average weight of mice in the silicosis group decreased significantly after two weeks of modeling (male: 20-18 g; female: 16-15 g), and then increased slightly (male: 20-18-21-23 g; female: 16-15-17-20 g). The results showed that sex had no significant effect on the multiple indexes of the pulmonary function at most time points (Figure 1B). The hematoxylin and eosin (H&E) staining showed a small number of minute silicotic nodules consisting of macrophages around the bronchus. The alveolus septum was thickened after two weeks of the silica stimulation in both male and female mice. By the fourth week, the volume and number of silicotic nodules increased, and some silicotic nodules were found to be merged. By six weeks, silicotic nodules were widely distributed in the lung tissues. The normal alveolus structure was destroyed and replaced by large numbers of silica nodules containing collagen. However, there was no significant difference in the area of the silicotic nodules between male and female mice, at all time points (Figure 2A–D).

### 2.2. Effects of Sex on the Collagen Deposition in Silicosis

**Figure 3:**
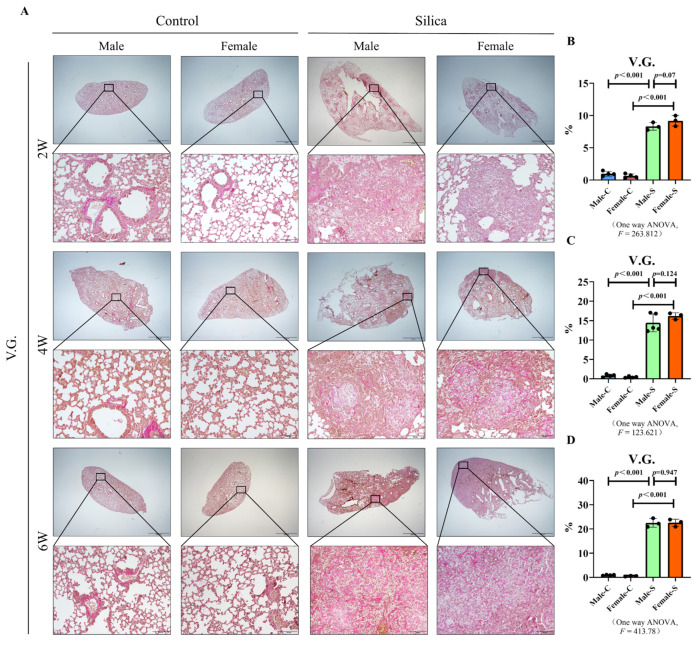
V.G. staining of the male and female mice exposed to silica in two, four, and six weeks, and the control groups. (**A**) V.G. staining of the lung tissues in mice exposed to silica (scale bar = 2000 and 100 µm). (**B**–**D**) Statistical analysis of the V.G. staining, *n* = 5 per group ((**B**)—2W, (**C**)—4W, (**D**)—6W).

**Figure 4:**
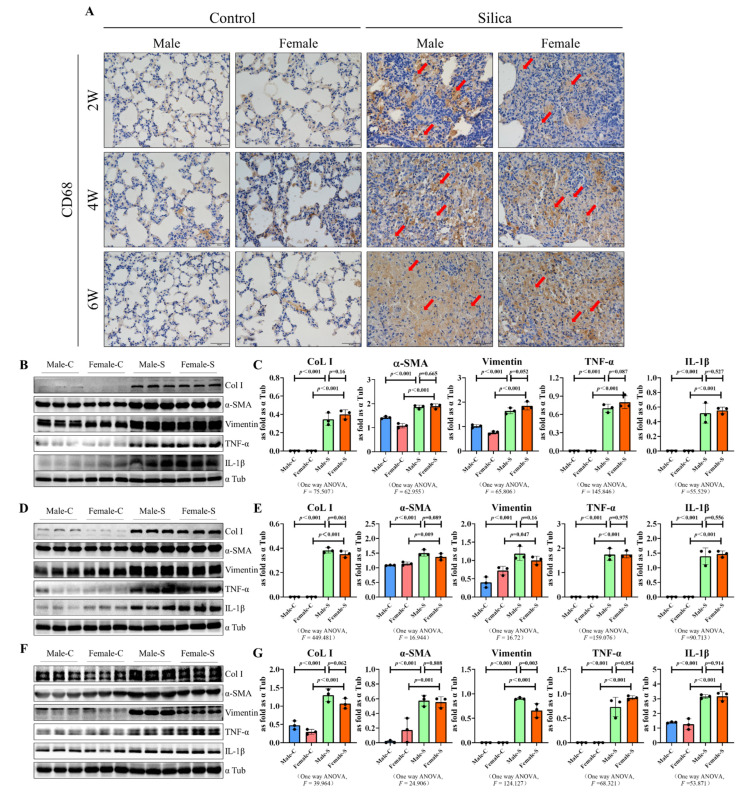
Western blot of CD68, COL I, vimentin, and α-SMA in male and female mice exposed to silica for two, four, six weeks, and the control groups. (**A**) Immunohistochemical staining was used to detect the expression of CD68 in the mouse lung tissues, arrow: CD68-positive cells (brown) (scale bar = 50 µm). (**B**–**G**) Expression levels of Col I, α-SMA, vimentin, IL 6 and IL 1β in the lungs of mice were measured using the western blot. Data are presented as the mean ± SD, *n* = 5 per group ((**B**,**C**)—2W, (**D**,**E**)—4W, (**F**,**G**)—6W).

The deposition of collagen is one of the main characteristics of silicosis. Van Gieson staining (V.G. staining) showed only a small amount of collagen around the blood vessels in the lung tissues of the male and female control groups. However, a huge amount of red-stained collagen was deposited in the silicotic nodules of the silica-exposed mice. In addition, we also found that the degree of collagen in the silicotic nodules increased in a time-dependent manner in both male and female silicosis groups. Fibrosis-related factors, type I collagen (COL I), and α-smooth muscle actin (α-SMA) [18,19] were analyzed using the western blot. No difference was observed in the expression of COL I and α-SMA between the female and male mice at two, four, or six weeks (Figure 3). These results indicated no difference in the collagen content between the female and male mice (Figure 3 and Figure 4).

### 2.3. Effects of Sex on the Inflammation Response in Silicosis

The immunohistochemical results showed that CD68, a recognized marker of the macrophages, was highly expressed in both silicotic male and female groups. These results indicated that silica activated the macrophages in both female and male mice. As evidence, the vimentin expression was also significantly increased in the silicosis model group. The expression of the inflammation-related factors interleukin 6 (IL 6) and interleukin 1β (IL 1β) were significantly up-regulated in the silicosis model group, but were consistent with the collagen deposition. There was no significant difference in the expression of these factors in the female and male mice, with respect to the markers or different time points (Figure 4).

### 2.4. Effects of Sex on the TGF-β Signal-Related Factors and Senescence-Related Factors in Silicosis

**Figure 5:**
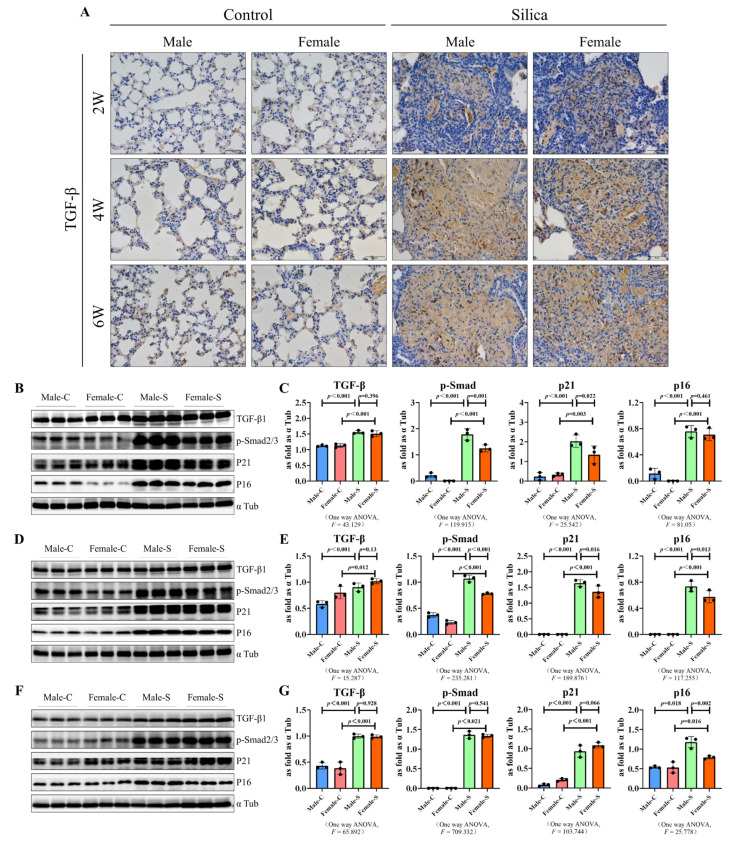
Western blots of TGF-β1, p-SMAD2/3, p21, and p16 in the male and female mice exposed to silica for two, four, six weeks, and the control groups. (**A**) Immunohistochemical staining was used to detect the expression of TGF-β (brown) in the mouse lung tissues (scale bar = 50 µm). (**B**–**G**) Expression levels of TGF-β1, p-SMAD2/3, p21, and p16 in the lungs of mice were measured using the western blot. Data are presented as the mean ± SD, *n* = 5 per group ((**B**,**C**)—2W, (**D**,**E**)—4W, (**F**,**G**)—6W).

Activation of the TGF-β signaling is one of the driving factors of pulmonary fibrosis [20], thus the effects of sex were also studied on the TGF-β signal-related factors in silicosis mice. The immunohistochemical results showed that TGF-β1 was highly expressed in the fibrotic lung tissues and there was no different between the males and females (Figure 5A). Even though the p-SMAD2/3 protein levels were significantly decreased at two and four weeks in females, than in the male mice, at six weeks, there was no difference in the expression of p-SMAD2/3 between the female mice. The role of the cellular senescence in the silicosis progression was described previously [4], we also detected the protein expression of senescence-related factors. Furthermore, the expression of p21 was slightly downregulated at two and four weeks. Interestingly, the p16 levels were significantly downregulated in the four and six week groups (Figure 5B–G).

## 3. Discussion

This study aimed to determine the effects of the sex differences in the lung responses after a single silica exposure, and the mechanisms of the differential susceptibility between sexes in silicosis. The cause of silicosis is fairly clear—the inhalation of quartz particles. The conventional wisdom is that only male miners would suffer from silicosis, with a perception that there are more men in industrial and labor workforces [21]. However, with deepening understanding of pulmonary diseases, the incidence of silicosis among jeans sandblasters and porcelain grinders, involving many women, have come to light [22,23]. Additionally, some areas with a high incidence of sandstorms, where people of both sexes and ages are chronically exposed to quartz particles and air pollution, are associated with the risk of developing or exacerbating IPF [24]. Based on the above data, it became necessary to study the effects of the sex differences in silicosis. To our knowledge, this is the first study to investigate the effect of sex differences on the lung’s response toward SiO_2_, in an animal model.

We observed that at all time points, the silica nodule formation and collagen deposition occurred in both female and male mice. However, consistent with our previous findings, the content of collagen in lung tissues, in all the groups, increased gradually with the exposure to silica and there was no difference between the male and female silicotic mice. Although it has been reported that the collagen content of both male and female mice gradually increases with age [25], our results clearly show that the increase of collagen in the lungs of mice in the model group is mainly caused by the stimulation of silica. Epidemiological results showed that the prevalence of the interstitial lung disease in children under two-years-old was much higher in males than in females [26]. These results may be attributed to their narrower airways, leading to a longer persistence of inhaled substances in the periphery of the male lungs. A more prolonged exposure may elicit a stronger adverse response and subsequent chronic disease [27]. On the contrary, another study showed that in rats, females were more sensitive to the bleomycin stimulation, compared with males. Female rats may have an exaggerated response to lung injury, relative to male rats, because of the female sex hormones, which have a direct fibrogenic activity on the lung fibroblasts [28]. However, studies in mice revealed that there was either no significant difference or greater male responsiveness in the bleomycin-induced pulmonary fibrosis [29]. The differential response to a bleomycin-induced pulmonary fibrosis, may be due to an inheritable trait controlled by a few genetic loci, and that the X-linked factor may be associated with the fibrosis phenotype [30]. In the present study, we found no difference in the silicotic nodule area and the collagen deposition, consistent with the results of the bleomycin-induced mice studies. Considering these results, we believed that the effect of sex on the formation of silicotic nodules was not strong enough to influence the area of silicotic nodules and the degree of collagen deposition. The pulmonary function of silicosis patients is significantly impaired [31], and the non-invasive whole-body plethysmograph system can obtain the pulmonary function data of mice well [32]. With reference to the previous literature [33], in the present study, due to the increased restrictive ventilatory dysfunction and airway resistance, the indexes of Penh, EF 50, PAU and PEFb were higher in silicotic mice. Moreover, between male and female silicotic mice, however, there was no difference in the pulmonary function, on the whole.

The molecular mechanisms of the silica-induced inflammation and fibrosis, have not been completely understood yet. However, there has been a generally accepted concept supporting the contribution of inflammation in the development of fibrosis and the autoimmune responses, due to silica inhalation [34,35]. Our previous studies also indicated that as an important part of the body’s defense against external damage, the inflammatory response also plays an important role in the progression of silicosis [5,7]. Normally, sex hormone receptors are typically expressed intracellularly, however, in immune cells, the expression of the sex hormones has also been found on the cell surface where they interact as part of the immunological synapse, during the antigen presentation [36]. Most studies have not taken these factors into account when summarizing the effects of sex hormones on immune cells or their responses [37]. Moreover, in most fields of silicosis and inflammation-related studies, the sex factor was not taken into account, they directly used male rats or mice [38]. However, based on our results, the oversight did not affect the final results, and there were no significant differences in the expression of the inflammation-related factors in female and male mice. As evidence, in a study by Du et al., female mice were used to study the inflammatory response in the silicosis progression [39]. It is worth noting that another study figured out that silica-exposed males had more lung inflammation, bronchoalveolar lavage fluid cells, IL-6, and autoantibodies [40]. The reason for this phenomenon may be the differences in mouse species.

TGF-β1 drives the activation and functions of the fibroblasts and myofibroblasts to mediate fibrosis [41,42,43,44]. The present study showed that the expression of TGF-β1 was slightly upregulated in female and male mice exposed to silica, consistent with the previous research. The downstream factors of TGF-β1 and SMAD2/3 were also upregulated. Different from the sex chromosomes, which alter the intrinsic nature of cells, hormones are extrinsic factors that coordinate the cell behavior on a physiological scale. Particularly, the sex hormones directly regulate the members of the TGF-β signaling pathway [45]. Researchers have found that estrogen inhibits the TGF-β signaling by stimulating the SMAD2/3 protein degradation [46]. Our experimental results were a little bit inconsistent with this study. Compared with male mice, the expression of p-SMAD2/3 was downregulated in the female mice at two and four weeks, however, at six weeks, there was no difference between the expression of TGF-β1 in the females and males. A report on the diabetic nephropathy suggested that estradiol had an inhibitory effect on TGFBR1, however, there were no significant sex differences with regard to the renal function and fibrosis [47], consistent with our data. Another study found that the most important role of estradiol is disrupting the TGF-β signaling, rather than regulating its expression [48]. It is believed that estrogen has an inhibitory effect on the TGF-β1 expression, however, Nadja et al. showed that, during the progression of fibrosis, the estrogen induced the balance or imbalance between pro-fibrotic and anti-fibrotic signaling [47]. Therefore, whether estrogen plays a protective role in the progression of fibrosis through the TGF β signal pathway remains to be determined, in the present study, the expression of TGF-β1 and p-SMAD2/3 of female mice and male silicosis mice was ultimately not different.

Clinical follow-up studies have shown that long-term estrogen deficiency in women with ovariectomies as young adults would experience accelerated senescence phenotypes, once they are older [49]. Another research indicated that an estrogen deficiency and cellular senescence represented independent mechanisms in the pathogenesis of osteoporosis [50]. Yu et al. first identified the upregulation of the p21 in the osteocytic-rich area of ovariectomized mice [51]. Our previous findings showed that the senescence-related factors were up-regulated in silicosis mice [3]. In the present study, we found that, as compared to the two and four weeks, the expression of p16 was significantly down-regulated at six weeks in silicotic female mice. For the order of the differential expression of p21 and p16, it has been proposed that the effect of p21^CIP1^ may be limited to the onset of senescence, whereas p16^INK4a^ maintains a durable growth arrest. Although induction of p21^CIP1^ is important for the initiation of senescence, its expression does not necessarily persist in senescent cells [52]. Our results suggested that male silicotic mice were more likely to show a cellular senescence than female mice.

Research in occupational pulmonary diseases has focused on men’s health, which introduced challenges and potential biases in characterizing women’s risks in specific workplaces and diagnostic delays [53]. In recent years, some researchers have also turned their attention to women with occupational lung disease [54,55]. Therefore, this paper focused on the effects of the sex differences in silicotic mice, although a detailed understanding of the disease processes will only be possible when the effect of sex on the signal crosstalk is elucidated. Our results showed that part of the classical fibrosis-related pathways were slightly downregulated in female silicotic mice, compared with males, but the ultimate degree of fibrosis did not differ between them.

## 4. Materials and Methods

### 4.1. Materials

Silica (s5631; Sigma–Aldrich, St. Louis, MO, USA), type I collagen (CoL I) (ab34710; Abcam, Cambridge, UK), α-smooth muscle actin (α-SMA) (ab5694; Abcam, Cambridge, UK), vimentin (ab92547, Abcam, Cambridge, UK), TGF-β1(ARG56429, Arigo, Shanghai, China), p-SMAD2/3(8828s, Cell Signaling, Massachusetts, USA), p21(ab109520, Abcam, Cambridge, UK), p16(A0262, Abclonal, Wuhan, China), IL-1β (DF6251; Affinity Biosciences, Cincinnati, OH, USA), IL-6 (A0286; ABclonal, Wuhan, China), α-Tub (AF7010, Affinity, Cincinnati, OH, USA), Whole Body Plethysmography (WBP) system (Buxco Research Systems, Wilmington, NC, USA), H&E dye (BA4025, Baso Diagnostics Inc., Zhuhai, China), Image-Pro Plus 6.0 software package (Media Cybernetics, Inc., Rockville, MD, USA), V.G. dye (BA4084, BaSO Diagnostics Inc., Zhuhai, China), goat anti-rabbit or goat anti-mouse secondary antibodies (074-1506/074-1806; Kirkegaard and Perry Laboratories, Gaithersburg, MD, USA), ECL prime western blotting detection reagent (ZD310A; ZomanBio, Beijing, China), and SPSS 20.0 software (IBM Corp., Armonk, NY, USA).

### 4.2. Establishment of the Mice Models

All of the study protocols were approved by the Committee on the Ethics of North China University of Science and Technology (LX2019033) and complied with the US National Institutes of Health Guide for the Care and Use of Laboratory Animals. A total of 120 C57BL/6 mice aged four weeks were purchased from Vital River Laboratory Animal Technology, China, and maintained at 12 h light/12 h dark period with free access to food and water.

The mice were divided into four groups as follows: control male (*n* = 30) and control female (*n* = 30) and male (*n* = 30) and female (*n* = 30) treatment groups. Both the control groups were subjected to the tracheal perfusion with 50 µL of 0.9% normal saline, whereas both the treatment groups were treated with tracheal perfusion with a 10 mg silica suspension (50 µL), using a small animal laryngoscope [7]. The mice were sacrificed at two, four, and six weeks, respectively. Left lobe tissues were dehydrated and embedded in paraffin, while the right lobe tissues were stored at −80 °C for the western blot.

### 4.3. Non-Invasive Measurement of the Pulmonary Function

The pulmonary functions were measured in the conscious mice with the FinePointe WBP system, as per the manufacturer’s instructions. The pulmonary function index values were automatically detected every 2 s. The measurement parameters were set as follows: 8 min for the duration of adaptation, 1 s for the nebulization, 5 min for the response, and 1 min for the recovery. The main indexes included the tidal volume (Tvb), minute volume (Mvb), peak expiratory flow (PEF), enhanced pause (Penh), pause (PAU), and expiratory flow 50 (EF50).

### 4.4. H&E Staining

Paraffin-embedded lung tissue sections were deparaffinized and rehydrated. The H&E dye were then added, dropwise, to observe the histopathological morphology. The area of the silicon nodules were counted using Image-Pro Plus 6.0 software package and then homogenized by the total area.

### 4.5. V.G. Staining

The de-waxed rehydrated lung tissue sections were treated with an equal proportion of hematoxylin A and hematoxylin B compared, and further, the V.G. dye was added. The area of collagen fiber stained by the V.G. dye was counted by Image-Pro Plus 6.0 software package and then homogenized by the total area.

### 4.6. Western Blot Assay

The proteins were separated using SDS-PAGE and further detected using the western blotting as described previously [56]. Briefly, the membranes were treated with primary antibodies of COL I, α-SMA, vimentin, TGF-β1, p-SMAD2/3, p21, p16, and α-Tub, respectively, (diluted at 1:1000). Further, they were incubated with goat anti-rabbit or goat anti-mouse secondary antibodies (diluted at 1:5000) in a blocking buffer. The immunoblots were visualized using the ECL prime western blotting detection reagent. The results were normalized against the corresponding control.

### 4.7. Statistical Analysis

The statistical analyses were performed using SPSS 20.0 software. Two-group comparisons were made using an unpaired Student’s *t*-test, and for multiple-group comparisons, a Shapiro—Wilk test was used to check the distribution of our data, and anova was used if the data were normally distributed, and the nonparametric tests were used if the data were not normally distributed. The statistical significance was achieved when *p* < 0.05 at a 95% confidence interval.

## Data Availability

Not applicable.
